# Supplementary figures and images for: The Stress-Chip: A microfluidic platform for stress analysis in Caenorhabditis elegans
Source: PLoS One. 2019 May 1;14(5):e0216283. doi: 10.1371/journal.pone.0216283 (PMC6493750; doi:10.1371/journal.pone.0216283)

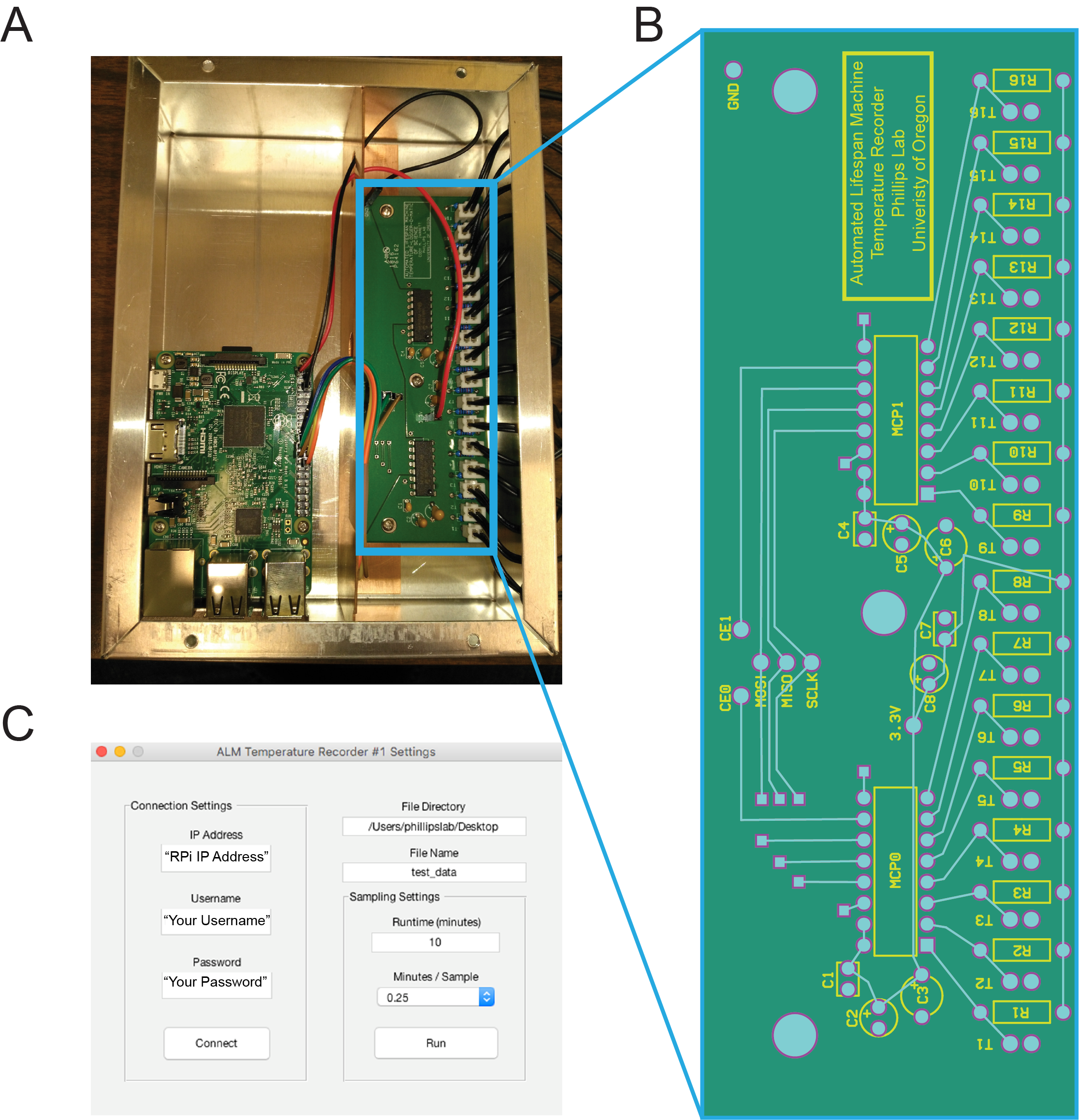

Supplement: S1 Fig — (A) The temperature recorder was built using a Raspberry Pi 3 single board computer, 16 thermistor probes, a custom printed circuit board and a custom case that provides separation between the analog and digital components for reduced electrical interference. (B) The custom PCB holds two 8 channel MCP3008 ADC chips. Signal noise was also reduced by inclusion of bypass capacitors C1-C3 and C4-C6. (C) A custom MATLAB GUI enables automated temperature experiments to be run at several discrete sample rates ranging from 2 samples/min to 4 samples/hour. (TIF) [file pone.0216283.s001.tif]

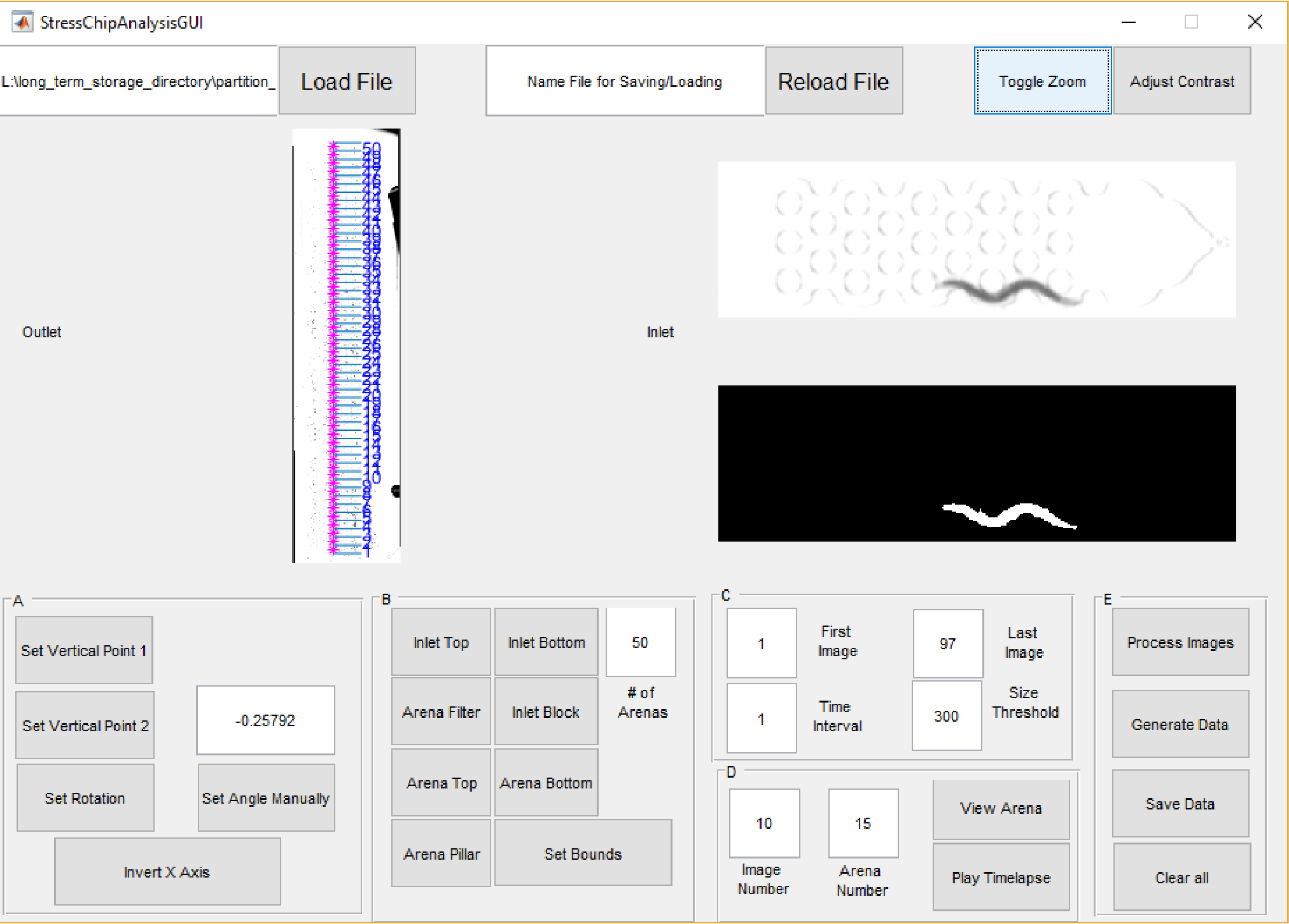

Supplement: S2 Fig — (A) Global image alignment pane. Using user set reference points, the interface corrects for any small deviations in the relative angle between chip and scanner. (B) Arena segmentation occurs after the user selects the bounds of the arena as well as number of arenas in each viewing frame. This also allows the user to exclude parts of the device from analysis that may have become clogged during operation as well as makes the interface adaptable between different variations of arena design. (C) Worm size and data range thresholding is set by the user and allows the exclusion of timepoints where image quality might have been lost through clogging. It also allows for the size threshold to be altered in case different stages or strains of worms necessitate changes in the image processing algorithm. (D) Individual worm viewing, and playback allows the user to check any single chamber at any timepoint in case there are any concerns about the worm inside or any discrepancies that require investigation. (E) The data review and export panel allows the user to view time-lapse movies of the data and displays both the unprocessed image as well as the computer vision output. In addition, once the user is satisfied with thresholding parameters, this panel is also used to direct the interface to generate and export the final data file with annotated worm deaths. (TIF) [file pone.0216283.s002.tif]
