# Supplementary material for: The Stress-Chip: A microfluidic platform for stress analysis in Caenorhabditis elegans
Source: PLoS One. 2019 May 1;14(5):e0216283. doi: 10.1371/journal.pone.0216283 (PMC6493750; doi:10.1371/journal.pone.0216283)
Supplement: S2 File — (ZIP) [file pone.0216283.s004.zip › S3_file/S3_File_UserGuide.docx]

**ALM 16-Channel Temperature Recorder Tutorial**

1. **Open MATLAB with Administrator Privileges**

**Mac OS:** Open an instance of the terminal by going to Go => Utilities => Terminal. In the terminal, type:

sudo open /Applications/MATLAB_R<version>.app/

<version> will be the year and letter of the MATLAB version on your system. For instance, 2016a.

**Note:** I don’t know for sure if versions of MATLAB older than 2016a will work with the temperature recorders, but as far as I know any 2014 or 2015 version will.

**Windows:** There should be a MATLAB icon on your Desktop from which to launch MATLAB. Right-click on this and click “Run as Administrator”. Otherwise, go to the MATLAB folder in your Program Files, find the MATLAB launcher .exe file, right-click it and select to run it as administrator.

1. **Install the Raspberry Pi Support Package**

**Mac & Windows:** With MATLAB open, click on the “Home” tab at the top of the IDE. Click the arrow of the “Add-Ons” icon in the toolbar. Then, click “Get Hardware Support Packages” at the bottom of the drop-down list. Make sure the “Install from Internet” bubble is selected in the window that opens, then click “Next”. Now, in the scroll window labeled “Support for:” on the left side, scroll down to “Raspberry Pi” and click on it. This brings up the two available support packages for Raspberry Pi in the window on the right. One requires MATLAB, whereas the other requires Simulink. Check the ‘Install’ box under the “Action” column for the one that requires MATLAB, but make sure the one requiring Simulink is unchecked (The Simulink support package is not necessary, and installing it will result in a longer install time). Click “Next”. You will be prompted to log into your MathWorks account. Do this and finish the installation.

1. **Install the .m Files**

If you haven’t already, download the .zip file we sent you that contains the .m files for using the temperature recorder. You can put these files anywhere on your system and use them from there, but I recommend setting up a dedicated folder for them in the “MATLAB” folder in your Documents directory (That way you can switch between working directories in MATLAB more quickly). Once you’ve set up a folder for the .m files (I recommend calling it something like “ALM16ChTempRecFiles”), extract the .m files into that folder.

1. **Set Up the Temperature Recorder**

All of the exposed ports on the recorder box are to the Raspberry Pi. First, connect an Ethernet cable between the Pi and the host computer that you will be using MATLAB on. Next, turn on the Pi.

**Note:** The Raspberry Pi doesn’t have a power button or switch like desktop/laptop computers. To power it on, just plug the micro-USB power supply into the Pi’s micro-USB port. Every time you turn it on, give it at least 1 minute to fully boot before you connect to it.

1. **Open the GUI**

In the “Current Folder” window on the left side of the MATLAB IDE window, double-click on the folder you set up with the temperature recorder .m files to make it the working directory. To open the GUI for using the temperature recorder, type the filename in MATLAB’s interpreter and press enter (E.g., for temperature recorder #1, you would type “B1ALMTemperatureRecorderGUI”, without the quotation marks).

Note: Another thing that can be done that appears to launch the GUI functionally, but doesn’t, is double-clicking the GUI .fig file. If this is done, the GUI appears but doesn’t work. So make sure you just enter the GUI file name in the interpreter.

1. **GUI Description**

Each recorder’s Pi has an IP address, username and password that can be pre-entered into the GUI for that recorder using MATLAB’s “Guide” function, so you should not need to change those fields after modifying the original MATLAB .fig file. Each recorder should be given a username “Your Username” and a password “Your Password”. The program that the GUI controls will sample data for a specified amount of time (“Runtime”) at a specified period (“Minutes / Sample”). Then it stores the data in a .csv file and puts the file in the directory specified in the “File Directory” text box with the name specified in the “File Name” textbox. Here is an example of what the GUI looks like when it’s completely set up and ready to begin sampling:

**
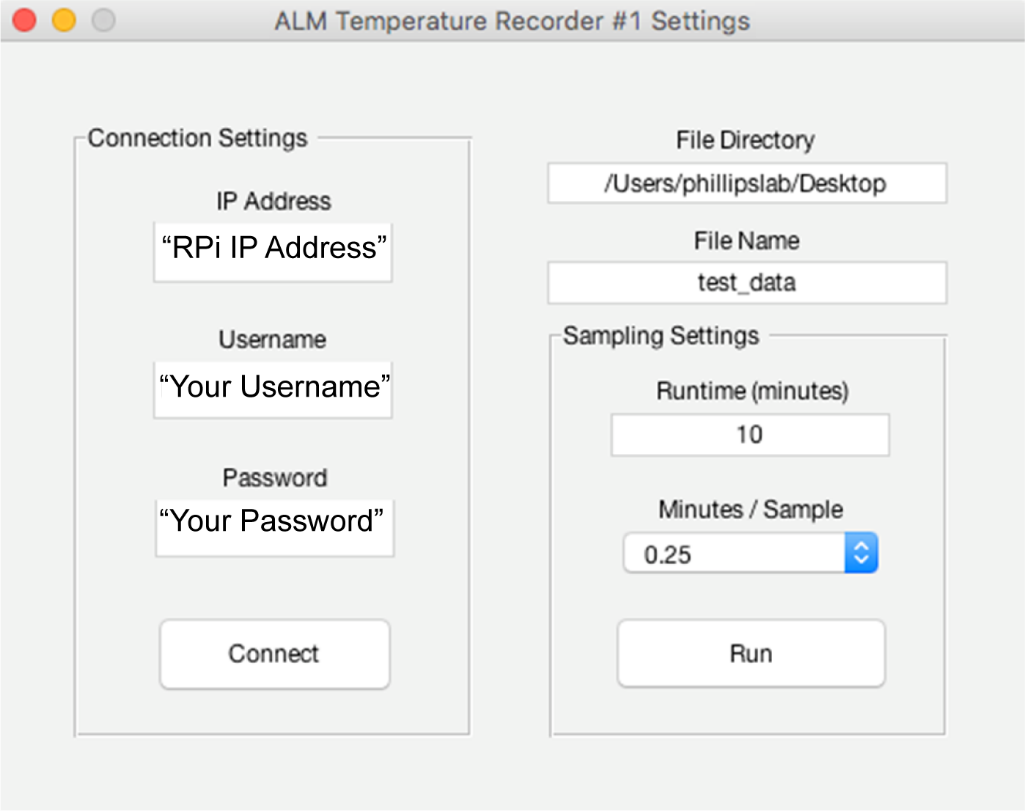
**

There are four sampling period options: 0.25, 0.5, 1 and 2. In the example image, it’s set to run for 10 minutes and will sample every 0.25 minutes. If you would like the recorder to run for hours or days, simply calculate the number of minutes in that amount of time and enter that into the “Runtime” field.

1. **GUI Use**

The first thing to do is make a connection to the Raspberry Pi. To do this, click the “Connect” button in the GUI.

Note: For some reason, the first connection attempt will always fail (At least if you’re using a Mac; I don’t think this happens with a PC). That’s okay. Give MATLAB about 3 minutes to respond. MATLAB will eventually throw some errors in the Command Window of the IDE saying that it failed to make the connection, like this:


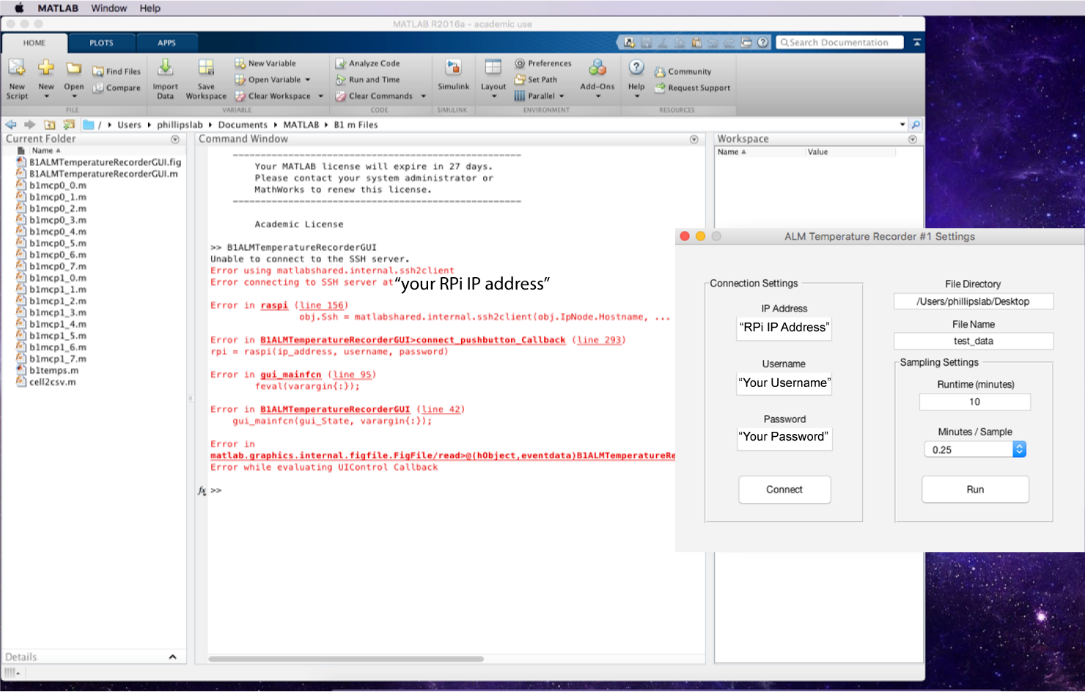


Once you get the error, just click the “Connect” button again and it should connect successfully. You will know that it does if this pops up:


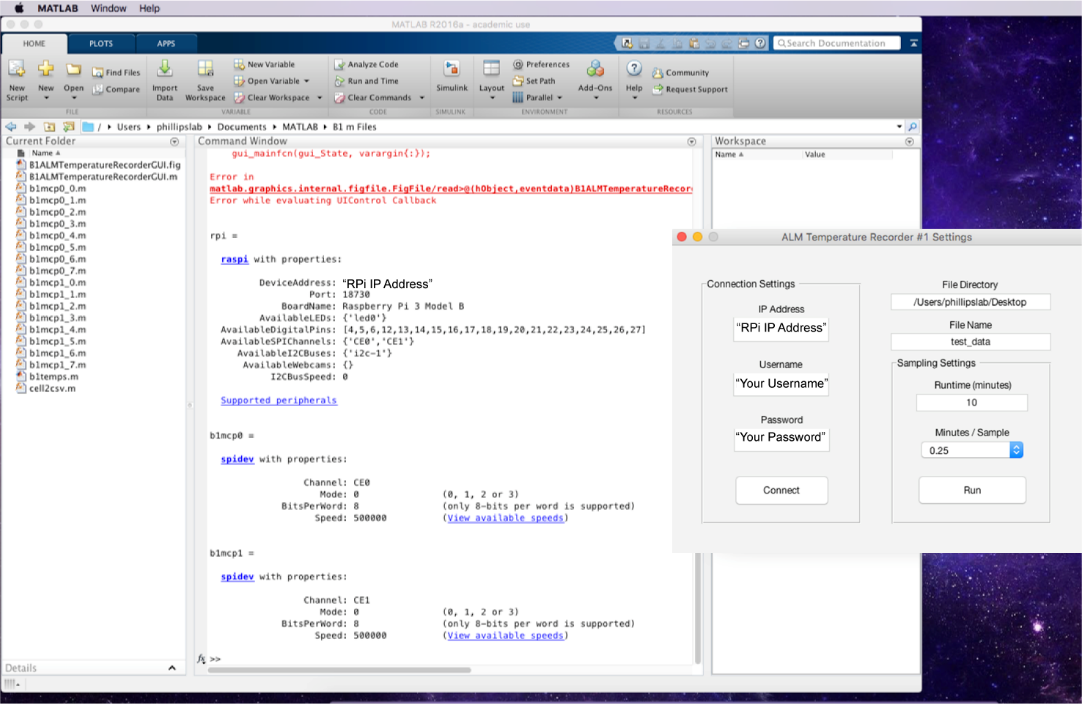


After the connection has been made, specify the last four parameters. When you’re ready to begin sampling, click the “Run” button.

After you click “Run”, the program will determine how long it takes to run the temperature sampling script on your machine, then it will display that time, then it will begin sampling. It will tell you when the sampling has begun and when it has ended. All of that looks like this:


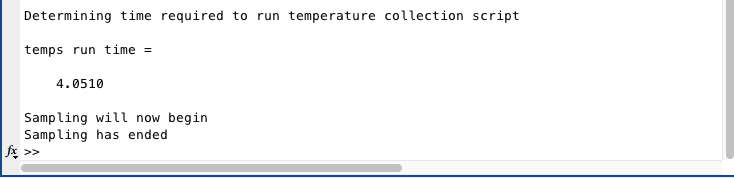


1. **When Recording Has Finished**

When the program is finished sampling, first close the GUI. Next, close MATLAB. Finally, unplug the power supply from the Raspberry Pi (You don’t have to issue a command to the Pi to shut down before unplugging it).

Note: You can initiate another sampling run if you do so within a couple of minutes of the previous one. To do this, just click “Run” again. But if you wait too long, for some reason the connection times out and MATLAB will not continue to work with the Pi. In that case, you must close MATLAB and turn off the Pi, then re-open MATLAB and plug the Pi back in. This timeout problem won’t occur after you first make a connection to the Pi, before you have done any sampling in that session. So after making the connection, you have plenty of time to enter the sampling settings and file information before you begin sampling. Also, if you accidentally close out the GUI but wanted to keep sampling, just re-open the GUI, set the parameters, and click “Run” to begin sampling again. The connection will remain even if the GUI gets closed. It only goes away if the Pi gets disconnected or MATLAB gets closed.
